# Endografts for the treatment of abdominal aortic aneurysms with a hostile neck anatomy: A systematic review

**DOI:** 10.3389/fsurg.2022.872705

**Published:** 2022-08-15

**Authors:** Christos Pitros, Pietro Mansi, Stavros Kakkos

**Affiliations:** ^1^ Department of Vascular Surgery, University of Patras Medical School, Patras, Greece; ^2^ Sapienza University of Rome, Rome, Italy

**Keywords:** abdominal aortic aneurysm (AAA), stent grafts, endografts, hostile neck, endovascular aneurysm repair (EVAR)

## Abstract

**Background:**

Endovascular aortic repair (EVAR) of abdominal aortic aneurysms (AAAs) has emerged as a better alternative to conventional open surgery for AAAs. The purpose of the review is to define the improvement in the clinical management of the patient with hostile neck AAAs due to the introduction of new endografts while giving a thorough description of their instructions for use (IFUs), main characteristics and part sizing, reporting their outcomes from clinical studies and categorizing their usability.

**Methods:**

A MEDLINE search was conducted using keyword-specific combinations. Clinical studies were searched *via* the clinicaltrials.gov website. Relevant articles' references were also hand-searched.

**Results:**

We retrieved 640 records describing Alto, Ovation iX, Treovance, Aorfix, Anaconda, Conformable, and Endurant II/IIs endografts. Aortic necks >60° can be managed with Anaconda, Aorfix, and Conformable, which can treat up to 90° necks requiring ≥15 mm (Anaconda ≥20 mm), and Treovance, which is eligible for necks ≤75° with ≥15 mm length. Ovation's innovation of combining polymer-filled O-rings with integral anchors can treat conical necked AAAs giving Ovation iX and Alto an advantage. Short-necked AAAs can be treated with Alto, eligible for necks as short as 7 mm, and Endurant II, which can treat ≥10 mm necks or 4 mm if used in conjunction with the EndoAnchors system, respectively. Alto and Conformable report a 100% technical success rate, absence of AAA-related death, migration, ruptures, and limb occlusion during follow-up. Endurant II and Ovation iX report >99% technical success rate and are almost free from the AAA mortality rate, ruptures, migration, and limb occlusion, while Ovation iX has a high rate of sac dilation (15.5%) in a 5-year follow-up. Anaconda is slightly better than Aorfix and Treovance, which are related to the lowest technical success rates, 98.3%, 96.3%, and 96%, respectively. Aorfix has the highest AAA mortality rate, 4% in a 60 month follow-up.

**Conclusion:**

Most new generation endografts described have comparable results. They broaden the eligibility of patients for EVAR due to their unique technical characteristics described. There is a lack of comparative studies for newer endografts and postmarket clinical studies with long-term results concerning the most recently approved devices described, Alto and Conformable.

## Introduction

An abdominal aortic aneurysm (AAA) is defined as a dilation to more than 3.0 cm in the infrarenal aorta. In men, the threshold for considering elective AAA repair is recommended to have ≥5.5 cm diameter, while in women with acceptable surgical risk, the threshold for considering elective AAA repair may be considered to have ≥5.0 cm diameter (1).

There are two main options for the treatment of AAA: open surgical repair and endovascular aortic repair (EVAR). Both options achieve a significant reduction in short- and long-term mortality (2).

Since endovascular aortic repair (EVAR) of infrarenal AAAs was first pioneered by Parodi et al. (3) and Volodos et al. (4) more than 30 years ago, EVAR has emerged as a less invasive alternative to conventional open surgery for the treatment of infrarenal AAAs; in fact, the application rate of EVAR and its clinical results have improved thanks to the evolution of stent-grafts and endovascular delivery systems (5).

Engineering developments in endovascular materials, along with the acquisition of improved technical skills by vascular surgeons and radiologists, have made EVAR results in patients with appropriate anatomy comparable to those of conventional open surgical repair (6). The most common factor precluding treatment with EVAR is AAA hostile anatomy and may involve not only the length and shape of the AAA neck but also other anatomical characteristics including thrombus and calcification of the landing zones. Therefore, neck anatomy is a major determinant of the suitability of patients for endovascular repair. Hostile neck anatomy (HNA) is nowadays assessed almost exclusively by computerized tomography angiography (CTA) scanning. In order to be labeled as hostile, the AAA neck has to have any of the following features: (1) >2 mm reverse taper within 1 cm below the renal arteries, (2) ≥60° angulation within 3 cm below renal arteries, (3) ≤10 mm neck length, (4) neck thrombus of ≥50% of circumference, and (5) >3 mm focal bulge in the neck (7). Others refer to the hostile aortic neck length as ≤15 mm (8, 9), with this length being the minimum requirement for most endografts.

All EVAR endografts stipulate anatomical criteria for treatment in their Instructions For Use (IFUs) (10).

Mostly common IFU criteria include infrarenal neck length of at least 10–15 mm, infrarenal neck diameter of 18–32 mm, infrarenal neck angulation <60°, and iliac access diameter of at least 6 mm. Other criteria frequently include limits on neck conicity, the volume of mural thrombus, and calcification. Patients with these criteria may be ineligible for open surgery due to comorbidities, and this is the reason patients may be treated with infra-renal EVAR outside of the recommended IFU in such cases. However, patients treated outside the IFU need a close follow-up, especially in the long term (11).

The safety and efficacy of these surgical procedures with unfavorable proximal neck remain controversial for conventional devices due to the inadequate sealing and the need for intraoperative or late endovascular adjunctive procedures. However, improvements in endovascular technology and the experience and expertise of endovascular specialists have recently modified the management of AAA patients with an improvement in perioperative outcomes and late results (12).

Nowadays, the endovascular treatment of AAAs in patients with severe proximal neck angulation is considered technically safe considering the use of newer endografts. Although angulation remains a feature that could increase intraoperative neck complications and require immediate adjunct neck procedures, one study reported that there is no significant difference in overall survival or the proportion of patients who remained reintervention-free at 5 years (13).

The purpose of the review is to to define the improvement in the clinical management of the patient with hostile neck AAAs due to the introduction of new endografts that are counter-angled, short, or conical AAA necks, traditionally defined as HNA.

## Methodology

**Figure 1:**
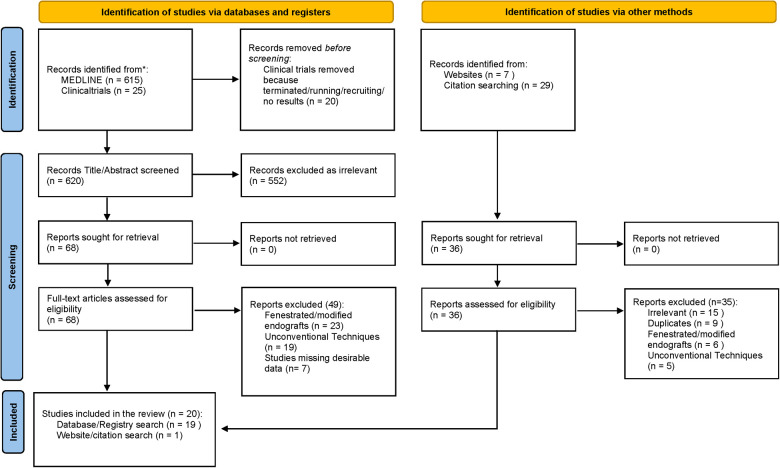
PRISMA flow chart.

A MEDLINE research using its PubMed interface was conducted (last search 25 December 2021); this retrieved 640 results from September 1994 to December 2021. The search strategy using keyword combinations is shown in Table 1. Also, a search for relevant previous or present clinical studies was made using the *clinicaltrials.gov* website. We performed a preliminary screening of each study's title and abstract and removed those that were irrelevant, before reviewing the full text of all potentially eligible clinical studies, on technical description or clinical results of endografts capable of dealing with HNA as depicted in the PRISMA flow chart (Figure 1). To be deemed anti-HNA in our study, an endograft must be able to resist >60 angled aortic necks, short aortic necks (<15 mm), conical (reverse taper) necks, or calcified/thrombosed necks. The references of relevant articles were also hand-searched. For the results section and tables, articles that describe the devices and IFU documents have been used to extract information and then combined to fully describe each endograft's main structural characteristics, IFU, and sizing. For the clinical results section and tables, 25 studies available for each endograft that provide at least 12 month follow-up outcomes have been used. To be included, the studies should also provide data about the outcomes summarized in Table 2 (follow-up period, technical success rate, secondary intervention, AAA-related/all causes mortality rate, endoleaks, ruptures, migration, limb occlusion, aneurysm sac diameter reduction/increase). Studies regarding fenestrated/modified endografts and/or using unconventional techniques were excluded.

**Table 1:** Search strategy of keyword combinations.

	Search strategy	Results
1	(EVAR OR “stent graft*” OR “HNA” OR “hostile neck” OR endografts OR AAA OR “Abdominal Aortic Aneurysm"[MeSH Terms])	31.275
2	(“stent graft*” OR endograft* OR EVAR OR endovascular OR alto OR anaconda OR conformable OR ovation OR aorfix OR treovance OR “endurant II*”)	779.154
3	(“hostile neck anatomy” OR “hostile neck” OR hostile OR angulated OR hyperangulated OR “short neck” OR “neck thrombus” OR conical)	39.732
4	(“Abdominal Aort*” OR “Abdominal Aortic Aneurysm” OR “AAA”)	60.923
5	thoracic[Title/Abstract]	
6	#1 AND #2 AND #3 AND #4	644
7	NOT #5	
8	#6 AND #7	615

*is a tool of searching for multiple word endings at once in databases.

**Table 2:** Endografts sizing of last-generation endografts capable of dealing with hostile aortic necks.

Device	Aortic body sizes								Iliac limb sizes			Aortic extension sizes		Iliac extension sizes*	
	Diameter(mm)			Length (mm)					Diameter (mm)	Length (mm)		Diameter (mm)	Length (mm)	Diameter (mm)	Length (mm)
Alto	20–34			80					14 Proximal 10–28	80–160		‐	‐	10–28	45
Anaconda	21.5–34			65					12 Proximal 10–23	80–180		19.5–34	40	Flared extensions	80–130
														10–17	
														12–23	
														Tapered extensions	
														13–23 Proximal	
														12–17 Distal	
														Straight extensions	
														10–18	60–140
Aorfix	24–31			81, 96, 111, 126					10–20 (Ipsilateral and Contralateral limb) 12 Proximal for contralateral limb	Ipsilateral limb	Contralateral limb	24–31	38	10–20	51, 82
										63, 80, 97	56–106 (7 sizes for any aortic body length)				
Conformable	20–36			120–200 (Overall including ipsilateral limb length)					12–14,5	120–200 (Overall including aortic body length)		20–36	45	10–27 16 Proximal	70–140
Endurant II	II	IIs	AUI	II	IIs	AUI			16 Proximal 10–28 Distal	82–199		23–36	49, 70	10–28	82
	23–36 Proximal 13–20 Distal (14 for IIs/AUI)			124– 166	103	102									
Ovation iX	20–34			80					14 Proximal 10–28	80–160		-	-	10–28	45
Treovance	20–36			80–120 (Overall including limb length)					14	80–120 (Overall including limb length)		20–36	40, 55, 70	(i) 8–14 (ii) 17–24 (i, ii) 15 Proximal	80–180 100–160
														Straight extensions	
														8–12	80

* Diameter of extension must be ≥ distal diameter of the iliac limb.

The technical features collected for each device were:(1) main characteristics (graft material, stent material, stent shape, radiopaque markers, sutures material, design, proximal fixation, fixation mechanism); (2) IFU (aortic landing zone length/diameter/angulation, iliac landing zone length/diameter, minimal access diameter of main/iliac body); and (3) endograft sizing (aortic body available diameters/lengths, iliac limb available diameters/lengths, aortic extension available diameters/lengths, iliac extension available diameters/lengths).

## Results

### Endograft description

#### Anaconda™ (Terumo Aortic)

Anaconda is a Terumo Aortic (Vascutek Ltd., Inchinnan, UK) AAA stent graft system approved by the European Union (CE mark) that consists of three components. The stent graft is built of ultrathin woven polyester fabric and a nitinol skeleton of a single-stranded nitinol wire that forms individual circular stents as it turns multiple times around it. It is topped with a dual-ring stent imitating the Anaconda snake. Numerous radiopaque tantalum markers are positioned throughout the prosthesis's length. The prosthesis is attached to the aorta through four pairs of nitinol hooks. The iliac legs are successively put into their positions (docking zones) with a 25-mm overlap. The primary body delivery method is a flexible thermoplastic fluoropolymer sheath reinforced internally with a very flexible metallic braided catheter shaft terminating in a tapered flexible top tip that improves the device's trackability *via* convoluted iliac arteries. The delivery system enables the operator to collapse, spin, advance/retract, and redeploy the main body. The contralateral body gate cannulation is assisted by a magnet system that utilizes a preloaded magnet wire to aid in the cannulation and deployment of the contralateral iliac leg. The redesigned Anaconda ONE-LOK has two extra mid rings in the body area and a universal diameter limb docking zone. The IFUs are described by the manufacturer as native proximal aortic neck diameters of 16–31 mm or 17.5–31 mm (using ONE-LOK) with a proximal aortic neck length of ≥15 mm. Anaconda is eligible for use for up to right angled (90°) aortic necks. Furthermore, a native iliac artery fixation length of ≥20 mm and a diameter of 8.5–21 mm are required (14–16).

#### Aorfix™ (Lombard Medical)

Aorfix™ is a Lombard Medical (Oxfordshire, UK) stent graft approved by FDA in 2013. The stent graft is a two-piece system consisting of (1) the main body incorporating an ipsilateral limb component and a contralateral socket and (2) a contralateral plug-in limb. It is mainly used for challenging neck anatomy as one of the few devices currently eligible for angulated necks up to 90°, thus broadening the patient eligibility for EVAR when first introduced. It employs woven polyester fabric and a continuous electropolished nitinol wire in comparison to older Z stents. Device's main characteristics are described in Table 3. Transrenal fixation of the four pairs of hooks (eight coplanar hooks) proximal on the graft is allowed when the implanted graft rings reform to a saddle-like “fishmouth” shape, and it has a small 8 mm fixation zone. The unique “fishmouth” shape of the proximal end, combined with the circular and the helical nitinol rings, gives this device an upper hand in conforming to tortuous anatomies. The ring stent configuration allows the device to apply better to the angle of the aorta without twisting or collapsing, and its “fishmouth” shape ensures a better seal while maintaining patency and luminal size. The device delivery is achieved *via* an 18-Fr outer diameter (OD) sheath for the main body and *via* a 16-Fr OD sheath for the contralateral limb. Aortic anatomical elements affecting aneurysm inclusion criteria include an infrarenal landing neck length ≥15 mm, an aortic neck diameter of 19–29 mm, and aortic neck angulation ≤ 90° for the proximal aortic device. Also, concerning the distal iliac landing zone, the iliac neck length is ≥15 mm and the iliac neck diameter is 9–19 mm (17–20).

**Table 3:** Main characteristics of last-generation endografts capable of dealing with hostile aortic necks (16, 20, 21, 27, 29, 33, 37).

Device	Graft Material	Stent Material	Stent Shape	Radiopaque Markers	Sutures Material	Design	Proximal Fixation	Fixation Mechanism
Alto	Polytetrafluoroethylene (PTFE)	Nitinol*	Z-shaped	Nitinol	-	Tri-modular	Suprarenal	Eight integral anchors and PTFE sealing rings
Anaconda	Woven polyester	Nitinol	Circular	Tantalum	-	Tri-modular	Infrarenal	Four pairs of nitinol hooks
Aorfix	Woven polyester	Nitinol	Spiral	Tantalum	Woven polyester	Bi-modular	Transrenal	Four sets of nitinol hooks (8 coplanar hooks)
Conformable	Expanded Polytetrafluoroethylene (ePTFE) and fluorinated ethylene propylene (FEP)	Nitinol	Z-shaped	Gold	-	Bi-modular	Infrarenal	Nitinol anchors and an ePTFE/FEP sealing cuff
Endurant II/IIs	Polyester	Nitinol	M-shaped (main body) Z-shaped (iliac limbs)	Platinum–iridium alloy w/ platinum “e” marker	Polyester and polyethylene	Bi-modular or tri-modular	Suprarenal	Anchor pins with optional EndoAnchors
Ovation	Polytetrafluoroethylene (PTFE)	Nitinol	Z-shaped	Nitinol	-	Tri-modular	Suprarenal	Integral anchors and PTFE sealing rings
Treovance	Woven polyester	Nitinol	Serpentine shaped	Platinum (90%) – iridium (10%)	Braided polyester	Tri-modular	Suprarenal and infrarenal	Suprarenal and infrarenal barbs

*Nickel-Titanium Alloy.

#### Conformable™ (GORE)

GORE® EXCLUDER® Conformable™ AAA endoprosthesis is used to treat infrarenal AAAs endovascularly. It is one of the most recent FDA-approved stent grafts obtaining its approval in December 2020. The endograft is a multicomponent system of bimodular design composed of a trunk-ipsilateral limb endoprosthesis and a contralateral limb endoprosthesis. In addition, in cases that require trunk or limb extension, an aortic extension endoprosthesis for proximal extension and an iliac extension endoprosthesis for distal extension are available. Each component's graft material is expanded polytetrafluoroethylene (ePTFE) and fluorinated ethylene propylene (FEP), which is supported along its external surface by a nitinol (nickel–titanium alloy) wire. At the leading (proximal) end of the trunk, nitinol anchors and an ePTFE/FEP sealing cuff are positioned, whereas a sealing cuff is located at the leading (proximal) end of the aortic extender. Each component has a gold radiopaque marking to facilitate identification. The endoprostheses are constrained on the delivery catheter using an ePTFE/FEP sleeve. The deployment mechanism has been updated to a three-step process, allowing the stent graft to be positioned up to three times prior to ultimate release from the delivery catheter. The initial step is to deploy the body and contralateral limb. A constricting loop around the graft's body enables the stent graft to be repositioned for level and orientation. The second step is to remove the constricting wire and loop (after a correct proximal position is confirmed). Third, the ipsilateral limb is deployed separately. The C3 system enables the proximal end of the endoprosthesis to be reconstrained following implantation, allowing the device to be rotated or shifted cranially or caudally as necessary. Repositioning the endograft may permit contralateral gate cannulation and placement closer to the lowest renal artery, thereby reducing the risk of inadequate sealing and subsequent complications. According to the IFU for this specific device, AAA patients with an infrarenal aortic neck diameter of 16–32 mm, a minimum aortic neck length of 15 mm, and a proximal aortic neck angle of up to 90° are eligible. In less severe angulation of up to 60°, the required minimum neck length decreases to a minimum of 10 mm. Iliac artery diameters between 8 and 25 mm and an iliac distal vascular seal zone length of at least 10 mm are also required (21–23).

#### Endurant II/IIs™ (Medtronic)

The Medtronic Endurant II™ (Medtronic Cardiovascular, Santa Rosa, CA, USA) obtained its approval in 2012. The main body is offered in two configurations: bifurcated Endurant II, which has a bimodular configuration, and bifurcated Endurant Iis, which has a trimodular configuration. Both configurations have suprarenal fixation and are composed of M/Z-shaped nitinol stents sewn to a polyester fabric graft. Additionally, the suprarenal stent has anchor pins to secure the stent graft inside the aorta above the renal arteries without blocking them with graft fabric. All stents on the ipsilateral limb are sewed to the outside of the fabric in the Endurant II bifurcated arrangement, resulting in a smooth inner lumen. The short form (Endurant Iis) enables the implantation of longer and more flexible targeted iliac limbs. Stents on the contralateral limb are sewed to the inside of the graft in all sizes. An Aorto-Uni-Iliac (AUI) main body is also available, enabling the choice of a two-piece graft configuration. The AUI device is indicated for the endovascular treatment of infrarenal abdominal aortic or aortoiliac aneurysms only in patients whose anatomy disqualifies the use of a bifurcated device. The proximal end of the limb configuration deploys within the limbs of the bifurcated configuration, while the distal end deploys into the iliac artery. The endograft part sizing is described in Table 4. If additional proximal or distal stent graft length is required, there are aortic and iliac limb extensions, respectively. The endograft's main advantage is the possibility to be used with the Heli-FX™ EndoAnchor™ system. When treating short infrarenal necks between 4 and 10 mm in length, the Heli-FX EndoAnchor system is necessary. The suggested minimum number of EndoAnchor implants for bifurcated endografts is determined by the native vessel diameter and is independent of the degree of endograft oversizing. For aortic neck diameters shorter than or equal to 29 mm, a minimum of four EndoAnchors is advised. For aortic neck diameters of 30–32 mm, a minimum of six EndoAnchors is advised. The Heli-FX EndoAnchor system utilizes a 16-Fr delivery system. However, there is a need for a bigger delivery system of 18–20 Fr concerning the main body. The iliac limb prostheses use 14 Fr at least. Aortic neck sizing with Endurant II accommodates necks ≥10 mm in length or ≥4 mm and <10 mm when used in conjunction with the Heli-FX EndoAnchor system, provided that the aortic diameter is between 19 and 32 mm and the angulation is ≤45°. As for the iliac bodies, iliac neck length of 15 mm and iliac neck diameter of 8–25 mm are needed (24–27).

**Table 4:** Clinical outcomes of endografts for hostile neck anatomy (16, 20, 21, 27, 29, 33, 37).

Device		Alto (46)	Anaconda (40)	Aorfix (17)	Conformable (21)	Endurant II (51)	Ovation iX (42, 43)	Treovance (52, 53)
Follow-up (months)		12	60	12–60	12	12–60	60	12
Technical success rate		100%	98.3%	96.3%	100%	99.3%	99.7%–100%	96%
Secondary intervention		2.7%	21.9%	1%–17%	2.5%	11%	7.6%–20.3%	3.5%–4.7%
Mortality rate	30-day	0%	1.7%	1.8% (median of groups)	0%	0%	0.3%–0.6%	0%
	AAA related	0%	2.3%	4% in 5 years follow-up	0%	0.8%	0.6%–0.7%	0%
	All causes	4%	34.1%	7%–31%	3.8%	17.7%	21.1%–21.7%	1.4%–6.4%
Endoleaks	I	1.3%	5.7%	0%–1% (combined with type III)	0%	0.8%	3.1% 4.9%–6% (combined with type III)	0.6%–1.5%
	II	48.3%	22.7%	9%–13%	43.6%	16.1%	10.5%–43%	15.3%–20.1%
	III	0%	N/A	0%–1% (combined with type I)	0%	0%	0.6% 4.9–6% (combined with type I)	0%
Ruptures		0%	0%	1%	0%	0%	0.6%	0%
Migration (>10 mm)		0%	1.7%	1–4%	0%	0%	0%	0%
Limb occlusion (thrombosis or stenosis)		0%	7.9%	1.83% only in year-1	0%	4.2% (12 months)	0.4%–4.3%	0.7%–2%
Aneurysm sac diameter	Reduction (>5 mm)	21.3%	32.4%	42%–61%	N/A	63.9%	52.4%–58%	46.3%–54.1%
	Increase (>5 mm)	1.6%	6.8%	1%–12%	1.5%	6%	15.1%–15.5%	0%–2.6%

* In this table, it has been a try to summarize clinical outcomes of different endografts from different clinical studies. Note that the studies have different patient selection criteria and may differ in follow-up periods; some patients may not have HNA, and others may have been treated outside the IFU (always a minority).

#### Ovation iX™ (Endologix)

Ovation iX™ is an Endologix Inc. (Santa Rosa, CA, USA) stent graft approved by FDA in 2012. This bifurcated device is of trimodular design with the main aortic body, iliac limbs, and the two (contralateral and ipsilateral) limb extensions. The Endologix Ovation stent graft is a low-profile endovascular device that was created to address the constraints of earlier stent grafts by fitting a broader range of iliac access and a wider variety of patients since it utilizes a 14-Fr hydrophilic catheter for the main aortic body and 13–14 Fr for the iliac limb prostheses. The 14-Fr catheter has the lowest profile of any catheter currently available. A network of inflated rings filled with a unique low-viscosity biocompatible liquid polymer that hardens during deployment provides proximal closure and support. The iliac limbs/extensions are made up of a PTFE-encased nitinol stent. Separate delivery catheters are packed with graft components. The device is placed, and the sheath is withdrawn during deployment. The proximal stent is subsequently deployed using the delivery handle's stent release knobs. An autoinjector is then used to provide the fill polymer *via* the fill connection port. After the fill polymer has been fixed, the delivery sheaths are removed. Prior to injection, three components of the fill polymer are mixed together. The components develop a radiopaque polymer once injected into the graft, fixing the sealing rings and channels in the aorta. Ovation's unique design is considered to provide a solution for conical (reverse tapered) necks since it can fixate the device proximally according to the form of the aortic neck. The device IFUs are described in Table 5. The endograft is indicated to treat AAAs characterized by a proximal neck length of ≥13 mm, a diameter of 16–30 mm, and an angulation of ≤60° if the proximal neck length is ≥10 mm. However, it is only indicated for proximal aortic neck angles ≤45° if the proximal neck length is <10 mm. As for the distal part, there it is indicated for an iliac length of ≥10 mm and a diameter of 8–25 mm (28–30).

**Table 5:** Instructions For Use (IFUs) of last-generation endografts capable of dealing with hostile aortic necks (16, 20, 21, 27, 29, 33, 37).

Device	Proximal aortic landing zone			Distal iliac landing zone		Access (minimum vessel diameter)*	
	Infrarenal landing neck length	Aortic neck diameter	Aortic neck angulation	Iliac neck length	Iliac neck diameter	Main body	Iliac limbs
Alto	≥7 mm	16–30 mm	≤60°	≥10 mm	8–25 mm	5 mm (15 Fr)	4 mm (12 Fr)
Anaconda	≥15mm	16–31 mm 17.5–31 mm (ONE-LOK)	≤90°	≥20 mm	8.5–21 mm	6.667 mm (20 Fr)	6 mm (18 Fr)
Aorfix	≥15 mm	19–29 mm	≤90°	≥15 mm	9–19 mm	6 mm (18 Fr)	5.333 mm (16 Fr)
Conformable	≥15 mm ≥10 mm when aortic neck angulation ≤60˚	16–32 mm	≤90°	≥10 mm	8–25 mm	5 mm (15 Fr)	4 mm (12 Fr)
Endurant II/IIs	≥10 mm or ≥4 mm and <10 mm when used in conjunction with the Heli-FX EndoAnchor system	19–32 mm	≤60°	≥15 mm	8–25 mm	6 mm (18 Fr) (5.333 mm (16 Fr) for EndoAnchors)	4.667 mm (14 Fr)
Ovation iX	≥13 mm	16–30 mm	≤45° if proximal neck length <10 mm ≤60° if proximal neck length ≥10mm	≥10 mm	8–25 mm	4.667 mm (14 Fr)	4.333 mm (13 Fr)
Treovance	≥ 10 mm	17–32 mm	≤ 60° if proximal neck length ≥10 mm ≤75° if proximal neck length ≥15 mm	For length of ≥10 mm an inside diameter of 8 mm – 13 mm or For length of ≥15 mm an inside diameter of 13 mm – 20 mm		6 mm (18 Fr)	4.333 mm (13 Fr)

#### Alto™ (Endologix)

The Endologix Alto™ (Santa Rosa, CA, USA) Abdominal Stent Graft System is an endovascular device delivered *via* a low-profile catheter to AAAs. The Alto endograft gained US FDA approval in 2020. As the successor of Endologix Ovation iX, it continues to be devoted to suprarenal fixation using integral anchors, PTFE sealing rings, and polymer-injected technology. Alternative self-expanding stents depend on radial force to achieve the proximal seal at the aneurysm neck. The polymer-injected technology represents a substantial shift from alternative self-expanding stents that rely on radial force to achieve the proximal seal at the aneurysm neck. The relocation of the sealing ring closer to the fabric's top edge, together with the included compliant balloon, intends to improve placement accuracy and reduce the indicated minimum aortic neck length to 7 mm. At the device bifurcation, additional webbing was added to balance the limbs and avoid contralateral limb prolapse during wire access and the docking limb's inner diameter was increased to 11 mm and was standardized across all device dimensions. Additionally, the aortic body limbs were offset by 5 mm to aid in limb identification, improving access to limbs. Device IFUs are reported as a proximal neck length of ≥7 mm, a neck diameter of 16–30 mm, a distal iliac landing zone with a length of ≥10 mm, and a diameter between 8 and 25 mm for the distal iliac sealing zone (31–33).

#### Treovance™ (Terumo Aortic)

Treovance™ is a Terumo Aortic (Bolton Medical Inc., FL, USA) stent graft system first approved by the FDA in 2015. The device is typically composed of a main bifurcated stent graft and two limb extension stent grafts, each delivered endovascularly *via* its distinct delivery system (Navitel Delivery System). Additional ancillary endovascular components such as cuff extensions are also available. In contrast to other devices available, it has the peculiarity of being a double (suprarenal and infrarenal) fixation endograft. The suprarenal barbs stay covered by the clasping mechanism prior to complete deployment of the main body, while the infrarenal barbs are buried in the valley of the first covered stent, allowing for safe cranial and caudal repositioning of the device until the proximal bare stent is released. This stent graft is composed of self-expanding serpentine nitinol stents that are sutured to a densely packed woven polyester fabric by braided polyester sutures. The stent scaffold is made up of interconnected sinusoidal springs. These stent springs are positioned evenly along the length of the graft fabric to give radial support and to allow the stent grafts to self-expand. All stent grafts are radiopaque to help with imaging and correct implantation. Cylindrical or tube-shaped radiopaque markers are used. Both are composed of a platinum–iridium alloy consisting of 90% platinum and 10% iridium. The main bifurcated stent graft features an exposed proximal stent with fixing barbs (suprarenal) to prevent migration. The second row of barbs is additionally inserted distally to the beginning of the covered area, roughly halfway through the first covered stent, to aid with infrarenal fixation. The main body is broadly divided into two limbs: the contralateral limb being 30 mm long and the ipsilateral limb being 60 mm long. Each of the iliac limbs can take limb extensions using the “Lock stent” mechanism, meaning that their stent contains dull barbs to engage the limb extension *in situ*. The Navitel delivery system has an outer profile of 18 Fr for main bodies or proximal cuffs of up to 28 mm diameter and 19 Fr for larger diameters. The delivery system used for the insertion of limb extensions has an outer profile of 13 Fr for distal diameters of up to 15 mm and 14 Fr for longer distal diameters. The anatomical inclusion criteria are described by the manufacturer as an infrarenal aortic neck diameter of 17–32 mm, with proximal aortic neck angulation of up to 75° with a minimum aortic neck length of 15 mm, in addition to the aortic neck angulation of up to 60° for aortic neck lengths longer than 10 mm. Furthermore, the iliac landing zone should be at a minimum length of 10 mm with a lumen diameter of 8–13 mm or 15 mm and an inner diameter of 14–20 mm (34–37).

### Clinical results

#### Aorfix

In the 2011 Arbiter 2 observational study (38), with a 12-month follow-up, the mortality rate was 3.3% aneurysm-related and up to 10% for all causes (6.6% 30 days postoperatively – not aneurysm related). The primary technical success was 93.3%, with assisted technical success being 100%, but without the need for open conversion. Despite that, five patients had local-vascular complications (16.6%) between the first 30 postoperational days, leading to the need for interventions. No type III endoleaks were described. However, two type I endoleaks were described: the first leading to persistent type I endoleaks and the second described as type II rather type I endoleaks in the 12-month follow-up. There were no aneurysm ruptures.

The PYTHAGORAS clinical trial (17) of the Aorfix endograft provided 5-year outcomes and is to date the highest quality clinical trial concerning the Aorfix stent graft, thus giving us a thorough report of the events 1, 3, and 5 years after the EVAR procedure. It has shown a technical success rate of 96.3% with a 5-year survival rate of 96%. There was a median 30-day mortality rate of 1.8% (1.5%, 0.9%, and 4.8% for groups of <60°, 60–90°, and >90° neck angles, respectively. The need for secondary interventions was about 1% in the 1-year, 14% in the 3-year, and 17% in the 5-year follow-up. The incidence of endoleaks was also described, with fewer type I and type III endoleaks, 1% combined and only in the first year in aortic neck angles ≥60°, and more type II endoleaks (13% in the 1-year, 8% in the 3-year, and 9% in the 5-year follow-up). The migration of stents in the first year was 1%, whereas Arbiter 2 reports no migration (38). Migration took place in 5% and 4% for the 3-year and 5-year follow-ups, respectively. Aneurysm sac shrinkage (>5 mm) and expansion (>5 mm) percentages were both increasing in each follow-up stage (1, 3, and 5 years after EVAR), being 42%, 57%, and 61% and 1%, 7%, and 12%, respectively. Five-year freedom from sac rupture was 99%.

A new 5-year large clinical trial of 500 participants, which is funded by Lombard Medical Ltd., is awaited to be completed in April 2022 (39).

#### Anaconda

The French EPI-ANA-01 Registry of Anaconda (40) is the biggest study considering Anaconda. In the cohort, there were 33.9% patients with HNA and 10.7% were treated outside the IFU. On a 60-month follow-up, Anaconda reports 98.3% primary technical success rate, with four aneurysm-related deaths. Secondary interventions were performed in 19.9% patients with a common cause of limb occlusion (7.9%) and endoleaks (I—5.7%, II—22.7%). The patients treated outside the IFU had significantly (*p* = .03) higher rates of migration, surgical conversion, and aneurysm sac expansion.

However, in a 100 patients Italian study between September 2005 and September 2008, the primary technical success rate was 100%, the same as freedom from aneurysm-related deaths in 24-month follow-up (41). Same technical success rate as in a 2008 Greek Single-Center Experience of a 51 patients cohort (15).

However, a 2014 study evaluating Anaconda against severe angulated infrarenal neck (>60°) of a 36 patients cohort shows a low technical success rate and primary clinical success rate, 83% and 78%, respectively, in 12-month follow-up, while there were no aneurysm-related deaths even in 48 months of follow-up (14).

#### Ovation iX

A retrospective analysis of the ENCORE database was made by Swerdlow et al. (42) in 2019, where a total of 1,296 patients were included in the analysis, from which 296 (23%) were monitored for 5 years. Combining the results with the Barleben et al. (43) report of the 5-year outcomes, summarized in Table 2, technical success was achieved (99.7%–100%) in long-term studies. Over the course of the study period, the 5-year freedom from rupture was 99.4%. Reinterventions were needed in 7.6%–20.3% of cases. Mortality rates related to the aneurysm were between 0.6% and 0.7% (21.1% and 21.7% of all causes). Type I/III endoleaks ranged between 4.9% and 6%, and type II endoleaks ranged between 10.5% and 43.4%. Freedom from stent migration was 100%, and freedom from occlusion was between 95.7% and 99.6%. As for the sac diameter outcomes, 15.1%–15.5% increased in size (>5 mm) and 52.4%–58% decreased. Only one patient (0.6%) had aneurysm rupture, the same percentage stated by Swerdlow et al. (42). In this 5-year study, 30-day mortality was 0.3%, and 1.6% of patients experienced a major adverse event within 30 days. Freedom from type IA endoleaks was 97.6% at 1 year and 97.1% at 3 years. Freedom from device-related reintervention was 96.2% at 1 year and 94.4% at 3 years. Freedom from sac expansion was 97.0% at 1 year and 90.3% at 3 years. Freedom from aneurysm-related mortality was 99.6% at 1 year and 99.5% at 3 years. In comparison, another study (44) with a median follow-up of 37 months reported a 2% 30-day mortality (not aneurysm related). Overall freedom from re-intervention was 96%. Freedom from type IA endoleaks was 100%. Freedom from sac expansion (>5 mm) was 2%.

Ovation systems use an autoinjector to inject a polymer filling into the sealing rings and channels to fix them on the aortic wall. Polymer leakage is known as the Achilles heel of the Ovation iX stent graft. A polymer leakage-specific review has shown that between November 2009 and August 2016, 26 individuals had polymer leakage from about 10,000 device implants (0.26%), of which 8 were reported in the USA, four were in Greece, and four were in Italy. Twenty-four individuals had anaphylactic symptoms, with hypotension being the predominant complaint due to anaphylaxis. There were no fatalities, and the aortic aneurysm was noted to be successfully excluded in 23 cases at the end of the procedure (45). In addition, three patients experienced intraoperative polymer leak (0.2%) in the retrospective analysis of the ENCORE database (42). All three experienced transient hypotension, including one requiring cardiopulmonary resuscitation.

#### Alto

The cause of polymer leaks of Ovation iX has been identified and addressed by Endologix in the next-generation Alto endograft. Additionally, more improvements have been made concerning the neck length and access to limbs, as described in the previous section. Results of the Expanding Patient Applicability with Polymer Sealing Ovation Alto Stent Graft (ELEVATE) (46) clinical study with a cohort of 75 patients have been promising. The study's follow-up period was 12 months. Freedom from all-cause mortality was 96%, with a 100% freedom from AAA-related mortality and 30-day mortality. Freedom from secondary interventions was 97.3%, while one patient had a type 1A endoleak and received ballooning and bare metal stent placement for intervention on postoperative day 48, which successfully treated the endoleak. Another patient had a device infection and was converted to open repair on postoperative day 273. Endoleak incidence was reported in 1.3% as type Ia and in 48.3% as type II. There were no type Ib, III, or IV endoleaks; also, there was no migration or limb occlusion, thrombosis- or stenosis-related, and no AAA ruptures were observed through 365-day post-treatment. The aneurysmal sac increased in diameter >5 mm in only 1.6% patients and decreased >5 mm in 21.3%. The fact that this clinical study was sponsored by Endologix is noteworthy; therefore, more clinical investigation of the device is advised.

A cohort study of 11 patients who underwent EVAR using Alto (47) reported the device's great potential in early outcomes, as it achieved 100% clinical success (no type I/III endoleak, sac enlargement, stent graft migration, polymer leakage, AAA related mortality, or secondary intervention) at 1- and 6-month follow-up.

Another small cohort study (31) describes the first seven patients treated with the Alto stent graft at a single center (Auckland Hospital, Auckland, New Zealand) from August 2016 to February 2017, also showing promising results. There were no secondary interventions, AAA-related deaths, migrations, or aneurysm increase in the 12-month follow-up period. There were two type II endoleaks at 1 month and one persistent at 12 months.

On 19 December 2021, a new clinical study was registered on *clinical trials.gov* named Hellenic Registry of Ovation Alto™ Abdominal Stent Graft System (48). The study will be conducted by Larissa University Hospital in collaboration with the University of Patras and University Hospitals of Crete and Alexandroupolis, all in Greece. The Ovation Alto™ Hellenic Registry is intended to expand the clinical knowledge base by collecting data on subjects treated with the Ovation Alto™ Abdominal Stent Graft System in actual clinical practice during the first postoperative year. It will be an observational study of a 60 patients cohort starting on 1 January 2021 with anticipated competition in December 2022.

#### Conformable

The AAA 13-03 clinical study of a cohort of 80 patients was conducted to assess the safety and effectiveness of the GORE^®^ EXCLUDER^®^ Conformable AAA Endoprosthesis in the treatment of infrarenal AAAs. There was 100% technical success in procedures, and only 2.5% of them needed secondary intervention. In this study, there has been no type I, type III, or type IV endoleaks. In addition, there has been no AAA rupture or migration. Of the patients, 43.6% have had type II endoleak and another 7.7% have had an indeterminate endoleak reported. Also, a sac increase of >5 mm was observed in only 1.5% through the 12-month window. The data were collected from the device IFUs (21).

In an initial clinical experience study (22) between November 2018 and June 2019, 12 patients were treated with the Conformable endograft. The absence of early proximal type I endoleak was reported. The endograft's strong conformability in the context of angulated necks was validated by the lack of substantial changes in aortic curvature between preoperative and postoperative analysis.

One of the main assets of the Conformable device is the repositionability featuring the C3 delivery system. A retrospective analysis of 2018 (49) showed that the repositionable GORE EXCLUDER when used in combination with the C3 delivery system had critical safety properties, including precise placement relative to the renal arteries and equivalent long-term efficacy.

A new clinical trial is awaited and much needed. Assessment of the GORE^®^ EXCLUDER^®^ Conformable AAA Endoprosthesis in the Treatment of AAAs study is estimated to be completed in December 2026 (50).

Because of the lack of clinical results using the device, further studies would be useful in broadening the picture of its advantages and disadvantages.

#### Endurant II

The premarket clinical study (51) was a prospective, multicenter, regulatory trial performed on a 150-patient cohort with follow-up of 60 months describing a technical success rate of 99.3% with an 11% need for secondary interventions. The all-cause mortality rate was 17.7% in the 5-year follow-up, but only 0.8% had AAA-related deaths. Type I and type II endoleaks were reported as 0.8% and 16.1%, respectively, with no type III endoleaks described. During the 12 months of follow-up, freedom from AAA ruptures and stent migration was 100% and freedom from aneurysmal sac enlargement (>5 mm) was 94%, while in 63.9% of the patients, aneurysm sac shrinkage (>5 mm) was observed.

A three-center cohort study on 79 patients (25) gave notable results about the short bifurcated version of the Endurant II stent graft (Iis). It has shown 98.7% freedom from all-cause 30-day mortality, and none of the cases were AAA-related. Also, the survival rate was up to 96% in the 12-month follow-up. Type II endoleaks were reported as 17.7% in the 30-day follow-up. Freedom from type I endoleaks was 96.6%, while 100% of cases were free from limb occlusion or device-related reinterventions.

A clinical study with patients treated with Endurant II/IIs in conjunction with Heli-FX EndoAnchor implants for short-neck AAA (26) gives promising outcomes. The patients were treated following the current recommendation of placing four EndoAnchors for necks of ≤29 mm diameter, whereas it is recommended to place six for necks >29 mm. Investigators reported an overall procedural success rate of 97.1% and a technical success rate of 88.6%. The duration of the EndoAnchor implantation was 17.1 ± 11.5 min. Through the 30-day follow-up, type IA endoleaks were reported in four patients, of which three resolved spontaneously by the 12-month follow-up. There was an additional type IA endoleak through the 12-month follow-up that has not resulted in AAA enlargement or required a secondary procedure. Freedom from all-cause mortality was 92.7% through the 12-month follow-up, with freedom from the secondary procedure of 95.4%. Given this, the Endurant II system has a significant ally against HNA.

#### Treovance

The Treovance stent graft is also a newer-generation device designed to improve deployment and fixation and increase applicability to more complex aortas with more sizing options; all these design improvements should ensure durability for better long-term outcomes. The unique aspects of the device include dual active proximal fixation (suprarenal stent and at the sealing stent) and a series of rounded barbs in the docking segments of the main body to prevent limb dislodgement. Early clinical studies with the Treovance abdominal endograft system in AAA patients (including HNA) demonstrated feasibility, but they were referred to small cohorts only. Further investigations confirmed the safety and favorable 1-year outcomes.

A multi-institutional, prospective, pivotal trial (35) (March–December 2011) was conducted at five European centers on 30 patients that have been monitored for 1 year after the procedure. There were 29 males (range 50–81); 27 patients had an isolated infrarenal AAA, whereas 3 patients (10%) had an associated common iliac artery aneurysm. The initial clinical experience with the Treovance stent graft, limited to the initial perioperative period, was satisfactory. The stent graft was delivered and deployed safely, even in highly angulated anatomies and through small, tortuous, or calcified accesses. There has not been any intraoperative type I or type III endoleak in this cohort of patients nor serious device-related adverse events after the implantation as well as no device-related deaths.

In the RATIONALE (52) (global postmarket registry for the Treovance stent graft 2018) postmarket approval registry evaluated, clinical success (96%), a secondary intervention rate (3.5%), reduction in the aneurysm sac size of 5 mm (40%), absent 30-day mortality, rupture migration of the endograft were accessed. After 1 year of follow-up, 8 (4%) reinterventions were required and 13 (6.4%) patients died, but none of these deaths was aneurysm-related.

One of the last multicenter prospective nonrandomized studies on 150 patients (53) found similar values compared to RATIONALE: successful aneurysm treatment at 1 year (93.1%) shrinkage of the aneurysm sac of >5 mm at 3 years (54.3%); the need for a secondary intervention has been estimated on 4.7% with the absence of mortality related to AAA. In May 2020, the Food and Drug Administration approved TREO based on the safety and effectiveness assessed at the 30-day and 1-year endpoints in the clinical trial. The trial continues and will provide longer-term results to determine whether the EVAR technology has improved sufficiently to sustain superiority beyond the early outcomes.

## Discussion

Our review has shown that even if the clinical results are comparable, the individual characteristics of the endografts are often based on different engineering structures and features to guarantee adaptation of various anatomy and needs, particularly in the case of HNA.

### Angulated necks (>60°)

Lombard Medical made a breakthrough by introducing Aorfix, which got FDA approval in 2013. It was the first third-generation device to address hyperangulated necks up to 90°. The unique “fishmouth” shape of the proximal end, in combination with the circular and helical nitinol rings, gives this device an upper hand in conforming to tortuous anatomies. The Anaconda device is also a device addressing the right-angled aortic necks. Its unique circular stent design has given it this ability. Bolton Medical Inc. got in the fight against >60° angulated necks 2 years later with their Treovance endograft. This stent graft is composed of evenly positioned serpentine nitinol stents that are sutured to a densely packed woven polyester fabric by braided polyester sutures. In addition to Treos novelty of being a double (suprarenal and infrarenal) fixation endograft to prevent migration, the stent scaffold is made up of interconnected sinusoidal springs giving it the ability of dealing with aortic neck angles up to 75° for neck lengths ≥15mm. The newest device treating necks greater than 60° is the Gore Excluder Conformable. The device is eligible for treating AAAs with proximal neck angulation of up to 90° because of its bifurcated design featuring a short main body with long limbs and its unique delivery system, C3, with the ability to reposition during the implantation procedure. This may be an additional useful feature for treating patients with HNA.

According to a Polish study on 100 patients (54), the most applicable device is Conformable (65%), while Aorfix reported one of the lowest feasibility rates (37%), below Anaconda (39%) and Treo (45%). In a study on 235 Greek individuals (55), greater rates of EVAR applicability were noted, with Aorfix being one of the last in the ranking (42.7%), once more near Anaconda (57.3%), while Treo showed better results (74.9%).

Aorfix seems to have a higher mortality rate than other available endografts, with the AAA-related mortality rate of up to 4%, while the secondary intervention rate was also high (17%) in 5-year follow-up (17). Limb occlusion incidents were comparable for Aorfix and Treovance in 12-month follow-up (∼2%), while Anaconda had 7.9% limb occlusion incidents in a 60-month follow-up (40). The Conformable endograft had a high rate of type II endoleaks (43.6%) but was free from other endoleak types, ruptures, migration, or limb occlusion incidents (21). Treovance was free from ruptures, migration, and AAA-related mortality and had comparable endoleaks and limb occlusion rates with the other endografts in 12-month follow-up (52, 53). Anaconda, Aorfix, and Treovance are well studied and have studies of quality reporting their clinical outcomes, unlike Conformable, which lacks big cohort studies and long-term follow-up.

### Conical (reverse taper) aortic necks

A conical neck is variously defined for each article concerning this type of HNA and is inextricably linked to type IA endoleaks incidence increase (56). A European retrospective multicenter study on 156 consecutive EVAR patients with short necks (<15 mm) indicates that a conical neck (reverse taper architecture with progressive neck dilation of >2 mm) is the single most powerful predictor of proximal failure in patients with a short proximal aortic neck treated with routine EVAR (57).

Endologix came up with a unique solution that only their Ovation system features. In addition to suprarenal fixation *via* integral anchors, a network of inflated rings filled with a unique low-viscosity biocompatible liquid polymer that solidifies during deployment provides proximal closure and support. An autoinjector is used to provide the fill polymer *via* the fill connection port. The O-ring polymer-filled system may have benefits but also comes with some drawbacks (58). The system's main advantages are reduction of endograft profile and no aortic neck stress due to endograft radial force, while its critical issues are polymer leakage and stability and durability of proximal endograft fixation.

Up to 4 years after the intervention, the Ovation stent graft device has been proved to be a safe choice for patients with both conventional and hostile anatomy who may fall outside the IFUs of other commonly used device platforms, even in patients with conical proximal necks (44). Thus, the Ovation system seems to be a fair choice for patients with conical aortic necks.

Both endografts seem to have low AAA mortality rates, with Alto surpassing Ovation iX in freedom from reintervention and sac expansion in 12-months of follow-up (42, 46). As we described previously in the review, Ovation iX has been associated with polymer leaks, a problem that the improved Ovation system—Alto—seems to address to date. In any case, the lack of long-term independent studies describing Alto's clinical outcomes deprives us of the ability to export safe conclusions while comparing the two generations of Ovation endografts for conical necks.

### Short aortic necks (<15 mm)

AAAs with a short neck pose a significant barrier to device eligibility, as the majority of endografts can be used in the range of 10–15 mm and only a small portion of them can treat proximal necks shorter than 10 mm.

The aforementioned devices Treovance, Conformable, and Ovation iX have the ability to counter short necks of <15 mm. An ≤60° angled neck is a prerequisite for Treovance and Conformable to treat necks of ≥10 mm, whereas Ovation iX can treat aortic necks at least 13 mm in length. From the devices described in our review, only Endurant II and Alto may address short necks of <10 mm.

The Endurant stent graft is a self-expanding nitinol device that is sewed utilizing an ultrahigh-molecular-weight polyethylene suture to improve stent-to-graft connection strength. Alto is made up of the following components: a network of inflated rings filled with a liquid polymer and iliac limbs/extensions (PTFE-encased nitinol). While the typical stent-nitinol graft's skeleton produces a constant radial force across the infrarenal neck, the Ovation device's polymer-filled sealing rings offer a gasket-like effect without radial force to assure closure (59).

For these devices to be eligible for use in patients with short aortic necks, even shorter than 10 mm that most new endografts for HNA can cover, they recruit unique features. Alto, an improved Ovation device, has upgraded the Ovation use eligibility for short necks to 7 mm due to the relocation of the sealing ring closer to the fabric's top edge and the included compliant balloon. The Endurant system has been directly associated with the the EndoAnchors system, giving an edge against HNA, which accredits Endurant for use even with aortic neck lengths as short as 4 mm. In addition, its manufacturer Medtronic came up with the patent of Endurant IIs bifurcated device, which enables the implantation of longer and more flexible targeted iliac limbs.

Alto and Endurant II have comparable results, with Alto performing better in the case of freedom from reintervention, limb occlusion, and sac enlargement, likely a result of different follow-up lengths (46, 51). Another endograft that is used to treat short necks with a minimum of 10 mm aortic neck length is the Nellix (Endologix) EndoVascular Aneurysm Sealing (EVAS) System. Due to high rates of mid- and long-term failure, the device has been withdrawn (60).

### Neck thrombus/calcification

Neck thrombus and neck calcification are conditions that are often outside the IFU, thus disallowing devices to be used. Neck thrombus can also be considered a risk factor related to thromboembolic complications (61). Also, it has been suggested that mechanical failure of intraluminal thrombus (ILT) could play a key role in the rupture of AAAs (62). Neck calcification of the abdominal aorta is not an uncommon finding, but no much literature is available about the role of aortic calcifications in AAA repair. It seems that calcification at the proximal neck of the aneurysm is related to major difficulties in fixation between the stent graft and the aortic wall. A new study based on a literature review (63) did find an association between neck calcification and reinterventions for complications, such as endoleaks, migration, and increased risk of thromboembolism. Endografts may claim promising features that could overcome these problems; however, in the actual scientific literature, there is a lack of comparison between the endografts related to this particular HNA condition, and further investigations are suggested.

### Limitations

Data for the study were collected and based to the discretion, experience, and methodology of each center and researcher; therefore, results should be interpreted with caution. We acknowledge the possibility of bias and questionable quality of results possibly originating from industry-driven research. Also, small cohort clinical studies and not randomized trials were used in the review. The clinical studies may have different median follow-up periods and patient selection criteria, including patients with a “normal” (i.e., not hostile) neck. Also, we cannot exclude that some procedures are done outside the IFU. We have to report there are no devices with the feature of treating calcified/thrombosed necks. We used predefined outcomes accepting the individual definitions of each study. We cannot exclude the possibility that our search has not missed any device capable of treating an HNA.

### Future work

Further randomized clinical trials or controlled clinical trials with high-quality evidence would be beneficial in assessing the performance of each one of the endografts described, all useful for HNA. A systematic review and meta-analysis that will compare the different early, mid-, and long-term outcomes and performance of each device available would be of great use.

## Conclusion

All new-generation endografts described in our review have comparable results. They broaden the eligibility of patients for EVAR due to their unique characteristics described. The use of each device should be personalized for each patient, taking the anatomical features into consideration. There is a lack of comparative studies for newer endografts and postmarket long-term clinical studies with significant results concerning the most recently approved devices described, Alto and Conformable.
